# Exploring the Emotional Pathway from Motivation to Facebook* Addiction in a Vietnamese Undergraduate Sample

**DOI:** 10.11621/pir.2024.0106

**Published:** 2024-03-15

**Authors:** Duy-Hung Le, Pham Quang Dao

**Affiliations:** a *Ho Chi Minh City University of Education, Vietnam*; b Ural Federal University named after the first President of Russia B.N. Yeltsin, Yekaterinburg, Russia

**Keywords:** motivation, Facebook* addiction, emotions elicited, reinforcement, punishment

## Abstract

**Background:**

Facebook* is one of the largest social media platforms in the world. The use of Facebook* can lead to several problems, such as Facebook* addiction. Previous studies have investigated the effects of reinforcing factors on problematic Facebook* use, but negative factors have been little studied.

**Objective:**

The objective of this study was to investigate the relationship between motivation and Facebook* addiction, and examine the influence of positive and negative emotions, which serve as reinforcement and punishment when using Facebook*.

**Design:**

A cross-sectional survey was conducted on 294 university students in Vietnam, with a mean age of 19.93 and a standard deviation of 1.27. The Bergen Facebook* Addiction Scale, the Scale of Positive and Negative Experience, and the Motives for Facebook* Use Scale were used to collect the data. SPSS 22.0 and AMOS 20 software were used for data analysis.

**Results:**

The results indicated that the students’ motivations to maintain relationships and pass time were positively associated with Facebook* usage, while motivations for virtual community, entertainment, “coolness,” and companionship did not significantly influence Facebook* usage. This use of Facebook* was found to elicit both positive and negative emotions. Both these emotions were associated with an increase in Facebook* addiction among the students.

**Conclusion:**

These results support the view that Facebook* addiction is positively associated with satisfaction with Facebook* use, and that both positive and negative emotions are associated with an increase in Facebook* addiction. Several limitations of the study are clarified.

## Introduction

Facebook* is one of the largest social networking platforms in the world, providing users with the ability to connect, exchange information, share content, and access a range of other features. For many, Facebook* has become an integral part of daily life, exerting a significant influence on the way users, particularly undergraduate students, live. A randomized investigation of 92 students in Hong Kong revealed that 46% of them frequently utilized Facebook* for daily study purposes, and 75% of them frequently used it for entertainment (Feng et al., 2019). Prior research has also suggested that Facebook* is a useful tool for teachers to assign homework and provide academic support for students (Aaen & Dalsgaard, 2019). Additionally, Facebook* fulfills a variety of user needs (Chen, 2019).

Using Facebook* has the potential to become addictive. While high-frequency Facebook* use has been linked to Facebook* addiction, it’s not accurate to equate excessive use with addiction (Ryan et al., 2014). For instance, an individual may devote considerable time to developing a personal Facebook* page to promote a product without becoming addicted. Facebook* usage is deemed addictive only when it becomes compulsive, and detrimentally affects both the users’ social interactions and personal functioning (Chakraborty, 2016). Reflecting on the criteria for addictive behavior, numerous scholars have identified six core elements of Facebook* addiction: salience, tolerance, mood modification, relapse, withdrawal, and conflict (Andreassen et al., 2012; Griffiths, 2005).

In recent years, the number of studies on this issue has increased dramatically. According to a meta-analysis that included 34,798 participants in 32 countries, the general prevalence of Facebook* addiction is 24%; among students, it is 23% (Cheng et al., 2021). The consequences of excessive Facebook* use are not only addiction but also many other problems, such as increased symptoms of depression, impairment of self-esteem and academic or work achievement, and negative effects on physical health and social relationships (Busalim et al., 2019; Feng et al., 2019; Ho, 2021; Nikbin et al., 2021).

The satisfaction of needs, including those for self-expression, independence, and relatedness, has been identified as a contributing factor to Facebook* addiction (Chen, 2019). A modeling study by Miranda et al. (2023) has shown that those who are motivated to use Facebook* for escape or social interaction are more likely to become addicted. These studies suggest that Facebook* addiction may stem from desires for satisfaction. This is consistent with the view of Skinner (1953) that behaviors which are positively reinforced tend to be repeated. In line with this assumption, when a Facebook* user receives satisfaction from using the platform, this satisfaction may promote the repetition of this behavior in the future. Skinner (1953) also noted that behaviors which are punished (generate negative emotions) tend to disappear. However, such punishment appears to have received little attention in the context of research on Facebook* addiction. Exploring this issue is a critical task that our study aimed to address.

Based on Cultural-Historical Activity Theory (Leontiev, 1987), which suggests that motivation is what leads to human action, and Social Learning Theory (Bandura, 1977), which suggests that previous reinforcements of a behavior can become motivations for people to repeat or stop certain behaviors, we designed the conceptual framework of the study.

### Motives and behavior behind using Facebook*

Many researchers argue that Facebook* use is not a passive behavior but rather a motivated one (Pornsakulvanich et al., 2008). According to the Uses and Gratifications Theory (Katz et al., 1973), users are fully aware of the needs which motivate them to seek and choose the media to satisfy them. However, there is opposition to the idea that users are not always aware of their reasons for using social media (Masciantonio & Bourguignon, 2023). Psychologists have found that there are many behaviors that an individual takes up without being aware of the motivation behind them, such as automatic behavior, habitualized behavior, unconscious actions, etc. (Lumer, 2019). The Uses and Gratifications Theory (Katz et al., 1973) seems to hold true in regard to motivating individuals to start using social media platforms such as Facebook*; however, it is diffcult to fully explain why they perpetuate and repeat such behavior.

To obtain a more comprehensive understanding of this issue, an alternative approach is necessary. According to Leontiev (1987), needs are the source of behavior. When a need encounters an object that has the potential to satisfy it according to the subject’s subjective point of view, motivation is formed. It is this motive that drives human behavior. While participating in an activity, the subject leaves marks on the objective world. This process is called externalization. The opposite of that is internalization, which is the process by which individuals incorporate their experiences from participating in an activity into their personal development. This experience can serve as a motivator for continued engagement or as a deterrent. Leontiev (1987) observed that individuals may not always be consciously aware of their motives. However, even in the absence of conscious awareness, the psychological processes underlying the person’s internal motivation continue to operate covertly during engagement in an activity. This theory has the potential to clarify what motivates and sustains Facebook* usage among users.

So, we hypothesized that motivations drive Facebook* usage.

### Use and emotions when using Facebook*

The emotions elicited during the use of Facebook* have been extensively studied by researchers. There is currently an opinion that different ways of utilizing Facebook* might have different emotional repercussions for individuals. In a critical review, Verduyn et al. (2017) proposed that active social media use can enhance subjective well-being, which encompasses both an individual’s emotional state and life satisfaction, through an increase in connections and social capital.

However, there is also research that has shown that active use of social networks may cause upward social comparisons and consequent negative moods among users (Hogue & Mills, 2019). Passive social media use can negatively impact subjective well-being by increasing upward social comparison and envy (Verduyn et al., 2017). Although passive social media use has been shown to reduce positive emotions through social comparison, it appears that these effects are mitigated in users with a strong sense of self (Cheng & Nhan, 2021). This means that if people have a strong sense of self, the negative emotional consequences of passive social media use appear to be inactive.

These pieces of evidence suggest that the relationship between social network use, specifically Facebook*, and the emotions elicited during its use is highly complex and influenced by numerous underlying factors. According to Cultural-Historical Activity Theory (Leontiev, 1987), to better understand this relationship, it is necessary to consider not only the individual’s internal psychological characteristics but also his or her cultural and historical context. While our study did not aim to fully resolve this complex issue, a hypothesis can be derived from the aforementioned studies that Facebook* use can elicit both positive and negative emotions in users, regardless of the type of their behavior on the platform.

Another factor to consider is the user’s level of Facebook* usage. Research by Labrague (2014) suggested that an increase in time spent on Facebook* is associated with an increase in negative emotional states. As such, this study utilized the Facebook* frequency variable as a key indicator of user behavior on the platform. Moreover, Leontiev (1987) also noted that emotions are a reflection of the relationship between an individual’s motivation and the outcome of their actions. Positive emotions arise when the motive and outcome align, whereas negative emotions arise when there is a discrepancy between the two. Based on these outlines, we hypothesized that Facebook* use can increase both positive and negative emotions in users.

### Emotions when using Facebook* and Facebook* addiction

According to Cultural-Historical Activity Theory (Leontiev, 1987), the process of internalization can facilitate the acquisition of experience and the development of personal insights. However, this theoretical perspective may be overly general and broad in scope. Consequently, our study adopted a social learning theory (Bandura, 1977) approach to examining the influence of the emotions elicited during Facebook* use on Facebook* addiction. It should be noted that the application of Social Learning Theory does not negate the validity of activity theory; rather, we argue that social learning theory can provide a more detailed explanation of certain issues that activity theory addresses at a more generalized level.

According to Social Learning Theory, past reinforcement and emotional punishment can serve as incentives for the repetition of a behavior (Bandura, 1977). This perspective is commonly applied in the analysis of alcoholism (Hittner, 1997) and may also be relevant to Facebook* addiction. Several studies have demonstrated that mood-enhancing incentives (positive reinforcement from a behavioral perspective) are associated with Facebook* addiction (Alzougool, 2018; Masur et al., 2014; Young et al., 2017). Cooper (2015) suggested that motivations such as avoidance and escape can be significant factors in the development of alcoholism. Recent evidence suggests that similar motivations, particularly those related to avoidance and escape, are also associated with Facebook* addiction (Masur et al., 2014). These findings support the role of social learning theory, which posits that past positive experiences and positive reinforcement may positively influence addictive behavior.

Despite the growing body of research on Facebook* addiction, the relationship between punishment (negative emotional experiences) and this phenomenon remains under investigation. Drawing on Social Learning Theory (Bandura, 1977), this study examines the role of positive and negative emotions, which act as reinforcement and punishment, as potential motivators for behavior change. Specifically, we hypothesize that positive emotions associated with Facebook* use may exacerbate symptoms of Facebook* addiction, while negative emotions may mitigate them.

### The current study

According to classical Activity Theory (Leontiev, 1987), an activity comprises a series of actions towards an object. In the process of action, individuals utilize intermediary tools to achieve their objectives. A tool is anything that assists the subject in achieving the activity’s goal. A tool can also be an action if it carries its own objective (Leontiev, 1987). For instance, the use of Facebook* can be a tool if it is employed to attain other goals or motives, such as learning, communication, etc. However, it can also be an activity if the use of Facebook* has its own objective: *e.g.,* creating and maintaining an online persona, building connections on Facebook*, seeking feedback from others, etc. In that case, using Facebook* is not a tool to achieve other goals, but the act of using Facebook* itself is the goal and becomes a real activity. Therefore, in this study, we believe that, depending upon the interaction between the subject, object, and tools, the use of Facebook* can be both a tool of activity and an activity in itself.

Based on the above theoretical foundations, we assumed that motivation would drive users to use Facebook*. The use of Facebook* leads to positive and negative emotions, which act as reinforcement and punishment. Thus, positive emotions would be associated with increased levels of Facebook* addiction symptoms, and conversely, negative emotions would be associated with reduced levels of Facebook* addiction symptoms. Here are the corresponding hypotheses:

*Hypothesis 1a*: Relationship Maintenance has a positive impact on Facebook* Use.
*Hypothesis 1b*: Passing Time has a positive impact on Facebook* Use.
*Hypothesis 1c*: Virtual Community has a positive impact on Facebook* Use.
*Hypothesis 1d*: Entertainment has a positive impact on Facebook* Use.
*Hypothesis 1e*: “Coolness” has a positive impact on Facebook* Use.
*Hypothesis 1f:* Companionship has a positive impact on Facebook* Use.
*Hypothesis 2*: Facebook* Use has a positive impact on Positive Emotion.
*Hypothesis 3*: Facebook* Use has a positive impact on Negative Emotion.
*Hypothesis 4*: Positive Emotion has a positive impact on Facebook* Addiction.
*Hypothesis 5*: Negative Emotion has a negative impact on Facebook* Addiction.

## Methods

### Participants

A cross-sectional survey was conducted using an online Google Form. Lecturers from the faculties at Van Lang University and the Ho Chi Minh City University of the Food Industry were contacted to distribute adapted Vietnamese questionnaires to their students. These two universities were selected to ensure sample representativeness, as the Ho Chi Minh City University of the Food Industry is a public university, and Van Lang University is private. All participants were informed of the study’s purpose and content and voluntarily participated in the study. The questionnaire took approximately 10-15 minutes to read and complete. Data were collected anonymously, and participants had the right to withdraw from the study at any time without providing a reason. After excluding invalid responses (incomplete information, under-18 years old, non-Facebook* users), a total of 294 valid surveys was obtained.

As presented in *Table 1*, the majority of participants were students from Van Lang University (81.6%), with the remaining 18.4% from the Ho Chi Minh City University of the Food Industry. In terms of gender, 37.8% of participants were male and 62.2% were female, with ages ranging from 18 to 22 years old (M = 19.93, SD = 1.27). The sample included 4.1% freshmen, 54.1% sophomores, 21.1% third-year students, and 20.7% fourth-year students. Regarding religion, 69.4% of participants were nonreligious, 20.7% were Buddhists, 8.8% were Christians, and 0.7% followed other religions. In terms of academic achievement, 16% of students had average performance, 66% had good achievement, 16.7% had very good academic records, and 1.4% had excellent academic records.

**Table 1:** Demographic information of participants

Construct	Item	Frequency	Percentage (%)
Gender	Male Female	111 183	37.8 62.2
Age	18 19 20 21 22	12 152 37 32 61	4.1 51.7 12.6 10.9 20.7
University	VLU HUFI	240 54	81.6 18.4
Course	1 2 3 4	12 159 62 61	4.1 54.1 21.1 20.7
Religion	Non-religious Buddhism Christian Other religions	204 61 26 2	69.4 20.7 8.8 .7
Academic performance	Average Good Very good Excellent	47 194 49 4	16 66 16.7 1.4

*Note. N = 294*

### Procedure

#### Questionnaires

Facebook* addiction. To measure the students’ levels of Facebook* addiction, the Bergen Facebook* Addiction Scale (Andreassen et al., 2012) was utilized. This tool comprises six items that assess the six core symptoms of addiction: salience, mood modification, tolerance, withdrawal, conflict, and relapse. Participants rated each item on a 5-point scale ranging from 1 (very rarely) to 5 (very often), with higher overall scores indicating a higher level of Facebook* addiction symptoms. The Vietnamese version of the scale has demonstrated good validity and reliability (Ho, 2023). In this study, the scale exhibited good reliability, as indicated by a Cronbach’s alpha coefficient of .82.

*Emotions Elicited.* The Scale of Positive and Negative Experience (SPANE) (Diener et al., 2010) was modified to assess the emotions experienced by the participants while using Facebook*. This tool consists of 12 items that measure two main types of emotions: positive and negative. Six items assess positive emotions (*e.g.,* “I feel pleasure when using Facebook*;” “I feel happy when using Facebook*”), while the remaining six items assess negative emotions (*e.g.,* “I feel sad when using Facebook*;” “I feel angry when using Facebook*”). In this study, the Cronbach’s alpha for positive emotions was .91 and for negative emotions was .93, indicating good reliability.

Facebook* use. The frequency of Facebook* usage among participants was assessed using a single item, “How often do you use Facebook*?” Participants rated their usage on a 5-point scale, with response options ranging from 1 (rarely) to 5 (always).

Motives for using Facebook*. The Motives for Facebook* Use Scale (Sheldon, 2008) was employed to assess the students’ motivations for using Facebook*. This scale comprises 26 items that measure six motivational categories: 1) relationship maintenance (6 items); 2) passing time (4 items); 3) virtual community (5 items); 4) entertainment (5 items); 5) “coolness” (3 items); and 6) companionship (3 items). As no progressive version of the scale was available, the questionnaire was translated into Vietnamese using a two-step process. First, a graduate with a bachelor’s degree in English pedagogy translated the questionnaire into Vietnamese. Then, a proficient psychologist translated the Vietnamese version back into English. A comparison of the translations revealed only minor dialectal differences and minimal deviation from the original translation. In this study, the Cronbach’s alpha coeffcients for the six scales were .83, .73, .88, .85, .73, and .88, indicating good reliability.

### Statistical Analyses

We used SPSS version 22 to perform statistical analysis and describe the demographic characteristics of our research participants. To test the reliability of our scales, we applied the Cronbach’s alpha coeffcient. According to Zeller (2005), a coeffcient between .7 and .8 is considered acceptable, between .8 and .9 is good, and above .9 is excellent. We also performed a Pearson correlation analysis to examine the relationships between variables. To test our hypothesis model, we used AMOS version 20 software to perform path analysis. Our independent variable was motivations for using Facebook*; our mediating variables were Facebook* usage, and positive and negative emotions experienced while using Facebook*; and our dependent variable was Facebook* addiction. We used bootstrapping with 5000 samples to assess the significance of the paths in our model.

## Results

Descriptive statistics for the study variables are presented in *Table 2*. The mean score for the students’ Facebook* addiction was 14.47, with a standard deviation of 4.61. Positive emotions were reported to occur more frequently during Facebook* use (*M* = 3.06, *SD* = .71) than negative emotions (*M* = 2.30, *SD* = .84). The primary motivations for using Facebook* among the students were relationship maintenance (*M* = 4.11, *SD* = .56) and “coolness,” which can be interpreted as self-expression (*M* = 4.03, *SD* = .59). *Table 3* provides detailed information on the correlations between the motivations, Facebook* usage, emotions arising from Facebook* use, and Facebook* addiction among the participants.

**Table 2:** Descriptive statistics of study variables

Variable	Mean	Sth. Deviation	Minimum	Maximum
Facebook* Addiction	14.47	4.61	6.00	30.00
Emotions Elicited				
*Positive emotion*	3.06	.71	1.00	5.00
*Negative emotion*	2.30	.84	1.00	5.00
Motives for Using Facebook*				
*Relationship Maintenance*	4.11	.56	2.17	5.00
*Passing Time*	3.15	.77	1.00	5.00
*Virtual Community*	3.26	.91	1.00	5.00
*Entertainment*	3.66	.67	1.00	5.00
*Coolness*	4.03	.59	1.75	5.00
*Companionship*	3.21	.95	1.00	5.00

*Note: N = 294.*

**Table 3:** Correlation between study variables

Variable	1.	2.	3.	4.	5.	6.	7.	8.	9.	10.
1. Facebook* Addiction	_									
2. Positive Emotion	.50***	_								
3. Negative Emotion	.29***	.27***	_							
4. Relationship Maintenance	.29***	.36***	-.03	_						
5. Passing Time	.45***	.48***	.17**	.36***	_					
6. Virtual Community	.36***	.41***	.15**	.38***	.53***	_				
7. Entertainment	.31***	.52***	.05	.44***	.55***	.52**	_			
8. Coolness	.13**	.32***	-.06	.46***	.22***	.34***	.47***	_		
9. Companionship	.44***	.50***	.21***	.36***	.55***	.65***	.58***	.31***	_	
10. Facebook* Use	.40***	.27***	.10	.28***	.25***	.13*	.22***	.11	.16**	_

Note: N = 294. The Pearson’s correlation coeffcient was reported. ***. Correlation is significant at the .001 level (2-tailed). **. Correlation is significant at the 0.01 level (2-tailed). *. Correlation is significant at the .05 level (2-tailed).

**Figure 1.**
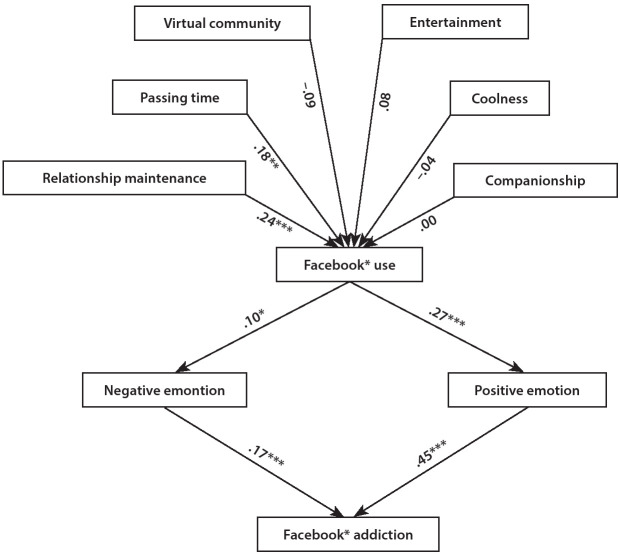
Research model analysis results

Note. * p .05; ** p .01; *** p .001.

The analytical data of the hypothetical model are presented in *Table 4* and *Figure 1*, with standardized coeffcients reported. This model is statistically significant (chi-square = 203.19; degrees of freedom = 19; *p* = .000). The results indicate that two motivations — relationship maintenance (*β* = 0.24; *p* = .001; 95% *CI* = [.107, .367]) and passing time (*β* = .18; *p* = .004; 95% *CI* = [.052, .317]) — had a statistically significant positive effect on Facebook* usage. However, incentives such as virtual community, entertainment, coolness, and companionship did not appear to have an effect on Facebook* usage (*p* > .05).

**Table 4:** Summary of the Hypothesis Model

Hypotheses	Path	*β*	*P-value*	95% CI		Result
				Lower Bound	Upper Bounds	
H1a	Relationship Maintenance → Facebook* Use	.24	.001	.107	.367	Supported
H1b	Passing Time → Facebook* Use	.18	.004	.052	.317	Supported
H1c	Virtual Community → Facebook* Use	–.09	.197	–.219	.044	Rejected
H1d	Entertainment → Facebook* Use	.08	.540	–.147	.291	Rejected
H1e	Coolness → Facebook* Use	–.04	.540	–.193	.108	Rejected
H1f	Companionship → Facebook* Use	.00	.922	–.153	.144	Rejected
H2	Facebook* Use → Positive Emotion	.27	<.001	.155	.372	Supported
H3	Facebook* Use → Negative Emotion	.10	.039	.004	.211	Supported
H4	Positive Emotion → Facebook* Addiction	.45	<.001	.350	.543	Supported
H5	Negative Emotion → Facebook* Addiction	.17	.001	.068	.273	Rejected

*Note: N = 294. Standardized coeffcients were reported.*

Furthermore, Facebook* use was found to be positively associated with both the positive emotions (*β* = .27; *p* < .001; 95% CI = [.155, .372]) and negative emotions (*β* = .10; *p* = .039, 95% *CI* = [.004, .211]) which arose when using the platform. Positive emotions were found to have a positive effect on Facebook* addiction (*β* = .45, *p* .001, 95% *CI* = [.350, .543]). Contrary to expectations, negative emotions were also found to have a positive association with Facebook* addiction (*β* = .17, *p* = .001, 95% *CI* = [.068, .273]).

## Discussion

The widespread use of social networking sites has provided tools to fulfill various human needs but has also led to complex mental health issues, including Facebook* addiction. Previous research suggests that need fulfillment may contribute to Facebook* addiction (Miranda et al., 2023). From a behavioral perspective, the fulfillment of users’ needs serves as positive reinforcement, promoting repeated Facebook* use and contributing to problematic behavior (Alzougool, 2018). However, punishment by negative emotions has received less attention, despite the hypothesis that it may limit future behavior (Bandura, 1977). Furthermore, the motivation for using Facebook* as a predictor of Facebook* use has received little empirical support, despite theoretical evidence. Based on the Cultural-Historical Activity Theory (Leontiev, 1987), this study also aimed to test this hypothesis.

First, we hypothesized that motives drive Facebook* use. Our results indicate that the motivations of maintaining relationships and passing time significantly and positively influence participants’ Facebook* usage. This is consistent with Cultural-Historical Activity Theory (Leontiev, 1987), which emphasizes the role of social and cultural factors in shaping human behavior. In this case, our findings suggest that Facebook*, as a cultural artifact, is being used as a tool to fulfill these social needs. However, the motivations of virtual community, entertainment, “coolness,” and companionship did not significantly influence the participants’ Facebook* usage.

Numerous studies have demonstrated that using Facebook* to maintain relationships is widespread, particularly when there is geographical distance (Billedo et al., 2015). Specifically, the motivation to maintain interpersonal relationships when using Facebook* has been found to be associated with repetitive Facebook* usage behavior that leads to addiction (Tang et al., 2016). Special and Li-Barber (2012) also found that maintaining relationships and passing time were the most common motivators of Facebook* usage among students.

As noted by Cultural-Historical Activity Theory, the context of an individual’s life is crucial for understanding human behavior (Leontiev, 1987). In today’s technological landscape, Facebook* is a tool that almost all students can easily access. Moreover, due to the impact of the COVID-19 pandemic, maintaining relationships or passing time on social networks has become more popular (Bowden-Green et al., 2021; Pahayahay & Khalili-Mahani, 2020). Additionally, it is important to note that university students are at an age when they often leave their families to continue their studies (Thurber & Walton, 2012). This is true in Vietnam, where the universities are mostly concentrated in large cities such as Ho Chi Minh City and Hanoi. Therefore, leaving family is a situation that many students face. Consequently, using Facebook* to meet the needs of maintaining communication and passing time is popular among students in Vietnam.

Second, the study results indicate a positive association between Facebook* use and both positive and negative emotions, thus supporting the second and third hypotheses. Previous studies have reported complex results regarding the effects of different types of Facebook* usage on emotions (Cheng & Nhan, 2021; Hogue & Mills, 2019; Verduyn et al., 2017). However, when considering the frequency of Facebook* use, this study suggests that using Facebook* can elicit both positive and negative emotions in users. This is consistent with previous research indicating that Facebook* use can be positively associated with both positive and negative emotions (Labrague, 2014; Nontasil & Payne, 2019). These findings support Leontiev’s (1987) Cultural-Historical Activity Theory, which posits that individuals gain experiences through the process of internalization during an activity — in this case, emotions.

Third, our study found a positive relationship between positive emotions and Facebook* addiction, supporting our fourth hypothesis. These results suggest that positive emotions may serve as a reinforcing agent, contributing to Facebook* addiction. This finding is consistent with previous research indicating that the receipt of positive reinforcement through motivation and escape from negative states is a crucial mechanism of addiction (Cooper, 2015).

But, contrary to our fifth hypothesis, negative emotions were also found to have a positive relationship with Facebook* addiction. We assumed that negative emotions would have a negative relationship with Facebook* addiction, as they would act as a punishment factor to limit the repetition of Facebook* use, as posited by Social Learning Theory (Bandura, 1977). This fifth hypothesis was not supported.

Our findings suggest that experiencing negative emotions while using Facebook* could also pose a risk of Facebook* addiction. Previous studies have posited that Facebook* usage can trigger negative emotions such as loneliness and frustration, which arise fromsocial comparison (Appel et al., 2016). In addition, other research suggests that frequent social comparisons on Facebook* might be linked to Face-book* addiction (Kim et al., 2021). The reason is that when users constantly compare their lives to others — for example, by viewing friends’ achievements, travel photos, or relationship updates — it can lead to feelings of inadequacy, envy, and so on. These negative emotions may prompt users to seek more validation and engagement on the platform, thus perpetuating the addictive cycle. Therefore, the negative emotions that arise from using Facebook* could lead to an increase in Facebook* usage, which may be a contributing factor to addiction. For instance, a person involved in an online argument may feel a great deal of exasperation but continue to use Facebook* to prove themselves.

Furthermore, research by Błachnio and Przepiorka (2016) has shown that an excessive focus on negative emotions may contribute to an increase in Facebook* addiction, while self-control regarding negative emotions can reduce the risk of addiction. Therefore, if users concentrate too much on their negative emotions while using Facebook*, this could lead to a heightened risk of addiction. In light of this research, we suggest that the ability to self-regulate emotions can be an important factor in resisting Facebook* addiction. These findings do not refute the concept of negative reinforcement but emphasize the need to consider an individual’s positive attributes when examining the mechanisms leading to Facebook* addiction.

In addition, there was the rather interesting result that positive and negative emotions were found to have a positive relationship in this study. This outcome can be interpreted to mean that while positive and negative emotions may not be directly related to each other, they could both be associated with Facebook* usage. The reason would be that using Facebook* is believed to potentially increase both positive and negative emotions.

## Implications

This study has several theoretical implications. The results help us to better understand the causes of Facebook* addiction from the perspectives of Cultural-Historical Activity Theory (Leontiev, 1987) and Social Learning Theory (Bandura, 1977). The motivation to use Facebook*, particularly the motivation to maintain relationships and pass time, played a significant role in promoting students’ use of Facebook*. This use of Facebook* led to various emotional experiences, which correspond to the process of internalization in activity theory. These experiences, when internalized, contribute to the motivation for repeated Facebook* use and can lead to addiction.

However, it is not always the case that positive reinforcement (positive emotions) promotes repeated Facebook* use, or that punishment (negative emotions) limits it. Instead, attention must be paid to the positive elements of the subject in the activity, such as the individual’s ability to self-regulate his or her emotions (Błachnio & Przepiorka, 2016). This result supports the use of Cultural-Historical Activity Theory (Leontiev, 1987) in analyzing human behavior.

Our study’s findings have practical implications for preventing and reducing Facebook* addiction. Our research indicates that the need to maintain relationships and pass time influences the amount of time spent on Facebook*. Therefore, to prevent Facebook* addiction, alternative methods of satisfying these needs should be explored, such as engaging in activities that improve social relationships, including joining clubs, or spending time with friends and family. Additionally, our research demonstrates that both positive and negative emotions are associated with Facebook* addiction. Addressing these emotional experiences may aid in preventing Facebook* addiction. Techniques such as mindfulness, which have been shown to reduce stress and negative emotions (Fogarty et al., 2015; Wu et al., 2022), may be encouraged. Contemplative practices, which have been found to generate positive emotions and increase awareness of social connections, may also be effective solutions.

Overall, these findings suggest that addressing the underlying motivations and emotional experiences associated with Facebook* use may be helpful in preventing addiction to the platform. It may be beneficial for individuals to find alternative ways to fulfill their needs and promote well-being outside of Facebook*.

## Limitations

This study has several limitations that warrant clarification. First, the data collection was conducted using online questionnaires via Google Forms, and the results were only obtained when participants submitted their responses. Consequently, the exact number of participants and the number of withdrawals are unknown. However, it should be noted that online responses may yield more honest feedback (Griffiths, 2010). Another limitation was the cross-sectional nature of this study, which precludes firm conclusions. Longitudinal studies are necessary to further develop the theoretical framework. Additionally, this study did not comprehensively investigate all user needs; for instance, the need to escape reality has been associated with Facebook* addiction (Young et al., 2017). Future studies may examine the overall impact of various needs and motivations on Facebook* usage.
